# Supplementary material for: Evaluation of ISSA proactive leading indicators for safety, health and well-being: Application of multi-criteria decision-making methods based on hesitant fuzzy
Source: PLoS One. 2025 Sep 2;20(9):e0322575. doi: 10.1371/journal.pone.0322575 (PMC12404562; doi:10.1371/journal.pone.0322575)
Supplement: S1 File — (DOCX) [file pone.0322575.s004.docx]

**Vision Zero Proactive Leading Indicators Questionnaire in Key Activities**

With regards

This questionnaire is designed to design a model "for evaluating proactive leading indicators of safety, health and well-being (SHW) based on the Vision Zero strategy of the International Social Security Association (ISSA): A case study in the petrochemical manufacturing industries of the Persian Gulf of Iran" which has been registered under research project number 23812 in the form of a master's thesis in HSE management at Shiraz University of Medical Sciences.

Your answer to these questions, dear expert, will help us complete the information and analyze the relevant information. It is hoped that these results will lead to the growth and development of safety in the country. Therefore, please help the implementers with your sincere cooperation to achieve the goals of this study.

I thank you for your attention, attention and patience in completing this questionnaire.

The approximate time to complete the questionnaire is 7 minutes.

Many thanks

Master's student in HSE Management

Vision Zero Proactive Leading Indicators Questionnaire in Key Activities (Part1: Quantitative Assessment)

In this method, the extent of implementation of key activities and whether they are implemented sufficiently, frequently and continuously is examined. Which is graded based on a five-point rating scale from "always" to "never".

**Enter your details**

**Petrochemical Company Name Place of Employment**

**Work Experience**

**Education**

Diploma

Associate Degree

Bachelor's Degree

Master's Degree

PhD

**Field of Study**

**Organizational Position**

**1. How often do leaders concretely show their commitment to integrating safety, health and well-being into work and behavioral processes?**

Well-being, physical and mental health and well-being in the workplace in terms of work organization are affected by psycho-social risks, for example, the type of job, workload and high work speed, regular work, lack of control over role ambiguity, role conflict, inflexible work schedule, and interpersonal relationships in the workplace, for example, risk of (conflict, harassment, bullying)

Mark only one oval per row.

Never or very rarely(0) Rarely(1) occasionally (2) (3) often always (4)

Safety

Health

Wellbeing

**2. How often, new leaders are selected based on intrinsic motivation with a proven track record in health safety and well-being?**

Mark only one oval per row.

Never or very rarely(0) Rarely(1) occasionally (2) (3) often always (4)

Safety

Health

Wellbeing

**3. How often are measures to reduce the risk of safety, health and well-being evaluated?**

Mark only one oval per row.

Never or very rarely(0) Rarely(1) occasionally (2) (3) often always (4)

Safety

Health

Wellbeing

**4. How often are unplanned health safety and well-being events followed up by leaders to review learning or improve health safety and well-being and provide feedback to those directly involved?**

Never or very rarely(0) Rarely(1) occasionally (2) (3) often always (4)

Safety

Health

Wellbeing

**5. How often is safety, health and well-being an integral part of employee induction processes?**

Mark only one oval per row.

Never or very rarely(0) Rarely(1) occasionally (2) (3) often always (4)

Safety

Health

Wellbeing

**6. How often are targeted programs and goals for improving safety, health and well-being evaluated?**

Mark only one oval per row.

Never or very rarely(0) Rarely(1) occasionally (2) (3) often always (4)

Safety

Health

Wellbeing

**7. How often is safety, health and well-being an integral part of the (important) discussion of pre-work briefings?**

Mark only one oval per row.

Never or very rarely(0) Rarely(1) occasionally (2) (3) often always (4)

Safety

Health

Wellbeing

**8. How often is the issue of safety, health and well-being systematically considered when planning and organizing work?**

Mark only one oval per row.

Never or very rarely(0) Rarely(1) occasionally (2) (3) often always (4)

Safety

Health

Wellbeing

**9. How often are technological and organizational innovations used to reduce risks and safety risks, health and well-being in the design phase?**

Mark only one oval per row.

Never or very rarely(0) Rarely(1) occasionally (2) (3) often always (4)

Safety

Health

Wellbeing

**10. How often are issues of safety, health and well-being considered in procurement processes (purchase of goods and services)?**

Mark only one oval per row.

Never or very rarely(0) Rarely(1) occasionally (2) (3) often always (4)

Safety

Health

Wellbeing

**11.How often are the concepts of safety, health and well-being included in the training for new hires?**

Mark only one oval per row.

Never or very rarely(0) Rarely(1) occasionally (2) (3) often always (4)

Safety

Health

Wellbeing

**12. How often are the concepts of safety, health and well-being included in retraining?**

**Mark only one oval per row.**

Never or very rarely(0) Rarely(1) occasionally (2) (3) often always (4)

Safety

Health

Wellbeing

**13. How often are the suggestions of employees to improve safety, health and well-being sufficiently followed up?**

Mark only one oval per row.

Never or very rarely(0) Rarely(1) occasionally (2) (3) often always (4)

Safety

Health

**14. How often are employees who have excellent safety, health and well-being performance encouraged?**

Mark only one oval per row.

Never or very rarely(0) Rarely(1) occasionally (2) (3) often always (4)

Safety

Health

Wellbeing

**Current status questionnaire**

Dear expert, please mark the following questions according to the existing situation from very high to very low with regard to aspects of safety, health and well-being.

What is the status of each of the following indicators in your company? If you are unsure about your answer, you can have more than one answer.

Mark only one oval per row.

very little few Medium a lot very much

Visible leadership commitment

Competent leadership

Evaluating risk management

Learning from unplanned events

Workplace and job induction

Define targets – develop programs

Pre-work briefings

Planning and organization of work

Innovation and change

Procurement

Initial training

Refresher training

Suggestions for improvement

Recognition and reward

**Desired status questionnaire**

Dear expert, please mark the following questions according to the desired situation, from very high to very low, according to the aspects of safety, health and well-being.

In your opinion, at what level should each of the following indicators be?

Mark only one oval per row.

very little few Medium a lot very much

Visible leadership commitment

Competent leadership

Evaluating risk management

Learning from unplanned events

Workplace and job induction

Define targets – develop programs

Pre-work briefings

Planning and organization of work

Innovation and change

Procurement

Initial training

Refresher training

Suggestions for improvement

Recognition and reward
