# Supplementary material for: Evaluation of ISSA proactive leading indicators for safety, health and well-being: Application of multi-criteria decision-making methods based on hesitant fuzzy
Source: PLoS One. 2025 Sep 2;20(9):e0322575. doi: 10.1371/journal.pone.0322575 (PMC12404562; doi:10.1371/journal.pone.0322575)
Supplement: Attachment — Submitted filename: Response to Reviewers.docx [file pone.0322575.s006.docx]

**Dear Dr. Ta-Chung Chu,**

Thank you for reviewing our manuscript submitted to your journal. The corrections requested by the esteemed reviewers are as follows:

**Reviewer #1:**

- According to the respected reviewer, the abbreviations in the title were removed.
- Abbreviations used in the abstract were revised.
- A list of abbreviations is provided in a table immediately after the abstract.
- All references cited in the text were reviewed and are available in the bibliography.
- The flowchart explaining the methodology was included within the main text to enhance the narrative coherence.
- Sensitivity analysis based on the weight of SHW indicators is included in the study in Section 2.3.6.
- Text formatting, including font and font size, was reviewed and edited throughout the document.
- Table 15 was edited.

**Reviewer #2:**

- The title of the study has been shortened and simplified to focus on the core objective of the study.
- Consistency in abbreviations was checked throughout the manuscript and typographical errors were corrected.
- According to the opinion of the respected reviewer, the introduction was reviewed and edited, and the requested items were added. An effort has been made to articulate the innovative aspects of this research separately.
- Formulas are numbered in the text.
- Detailed and lengthy calculations have been moved to the appendix.
- Thank you for the attention and care of the respected reviewer number 2, regarding the study methodology, which the respected reviewer has pointed out, no study has used multi-criteria decision-making methods to determine the importance, weight, and relationships between these indicators. For this reason, no information has been provided on how to use these methods in the field of the study. However, an effort has been made to clarify this at the end of the introduction, and relevant items have been added to the article text.
- In section 3.5, a comparison between IAM-VZPLI and EMA-VZPLI was provided and specific criteria were provided to evaluate which method is more effective in assessing the VZPLI.
- The discussion Broke down into clear sub-sections.
- Actionable management recommendations were provided at the end of the discussion.

**Reviewer #3:**

- The introduction section was edited based on the reviewer's comments and the requested items were added.
- According to the respected reviewer, the advantages of the evaluation method presented in the study were added to the introduction section.
- Comparison with other methods and studies was added to discussion section.
- The discussion Broke down into clear sub-sections.

There were similar comments from some reviewers, which were corrected and added.

Kind regards,

Jahangiri
